# An mHealth Self-management System for Support Children With Acute Lymphocytic Leukemia and Their Caregivers: Qualitative Co-design Study

**DOI:** 10.2196/36721

**Published:** 2022-04-15

**Authors:** Hamed Mehdizadeh, Farkhondeh Asadi, Hassan Emami, Azim Mehrvar, Eslam Nazemi

**Affiliations:** 1 Department of Health Information Technology School of Allied Medical Sciences Mazandaran University of Medical Sciences Sari Iran; 2 Health Information Technology and Management Department School of Allied Medical Sciences Shahid Beheshti University of Medical Sciences Tehran Iran; 3 MAHAK Hematology Oncology Research Center Shahid Beheshti University of Medical Sciences Tehran Iran; 4 AJA University of Medical Sciences Tehran Iran; 5 Department of Electrical and Computer Engineering Shahid Beheshti University Tehran Iran

**Keywords:** digital health, eHealth, mHealth, mobile app, smartphone, mobile phone, self-management, patient education, children, caregivers, acute lymphocytic leukemia, user-centered design

## Abstract

**Background:**

The unique features of smartphones have extended their use in different fields, especially in the health care domain. These features offer new opportunities to support patients with chronic conditions by providing them with information, education, and self-management skills. We developed a digital self-management system to support children with cancer and their caregivers in Iran (low- and middle-income country).

**Objective:**

This study is aimed at the development and preliminary evaluation of a cancer self-management system (CanSelfMan) tailored to the needs of children with cancer and their parents or caregivers.

**Methods:**

This study was conducted in collaboration with a multidisciplinary team between January and February 2020 at MAHAK’s Pediatric Cancer Treatment and Research Center. We developed a self-management system in six stages: requirement analysis, conformity assessment, preparation of educational content, app prototyping, preliminary evaluation, and developing the final version.

**Results:**

A total of 35 people (n=24, 69% parents and n=11, 31% children) volunteered to participate in the study. However, only 63% (15/24) of parents and 73% (8/11) of children were eligible to participate. By adopting a user-centered design approach, we developed a mobile app, CanSelfMan, that includes five main modules (knowledge base, self-management tips, self-assessment report, ask a question, and reminders) that provide access to reliable information about acute lymphocytic leukemia and the self-management skills required for side effect measurement and reporting. A web-based dashboard was also developed for oncologists and included a dashboard to monitor users’ symptoms and answer their questions.

**Conclusions:**

The CanSelfMan app can support these groups by providing access to reliable information about cancer, facilitating communication between children or parents and health care providers, and helping promote medication adherence through a reminder function. The active participation of the target group can help identify their needs. Therefore, through the involvement of stakeholders such as patients, caregivers, and oncologists in the design process, we improved usability and ensured that the final product was useful. This app is now ready to proceed with feasibility studies.

## Introduction

### Background

More than 90% of children worldwide live in low- and middle-income countries (LMICs) where accessing high-quality health care is difficult or expensive [1]. On the basis of the Global Cancer Inventory reports, by 2025, the number of new cancer cases will be higher in LMICs than in other regions [2]. Although the incidence of cancer is very low in children compared with adults, it is still the second leading cause of mortality among children, even in high-income countries [3]. It is predicted that, by 2030, approximately 60 million children will die worldwide because of cancer before the age of 5 years [4]. Iran is a developing country with a population of approximately 84 million. Similar to most LMICs, there are no precise statistics on childhood cancer in Iran [1]. The results of a systematic review showed that the incidence rate of pediatric cancer in Iran is 16.5 per 100,000, which is slightly higher than that in other countries [5]. Moreover, reports related to provincial registries indicate that leukemia (with a prevalence of 30%) is the most prevalent type of cancer among children in Iran, similar to other countries [6-8].

Approximately 80% of all the cases of leukemia in children are of the acute lymphoblastic type [9]. The common treatment for this disease is chemotherapy, which causes severe side effects [10-12]. The intensity of the symptoms and their effects on the child are sometimes so high that without the ability to control and manage these symptoms, they may lead to hospitalization or even withdrawal from the treatment plan by the patients and their families [13,14]. Furthermore, patient management at home is highly challenging for patients, families, and caregivers [15]. These challenges include the ability to manage complex therapeutic protocols (eg, diagnosis and measures to control the symptoms), proper nutrition diets, coping with psychological side effects, and interacting with the health care system during the treatment plan [16]. These challenges are especially complicated in children and adolescents with chronic conditions, and adherence to therapeutic protocols in this group is <50% [17]. Therefore, empowering patients by providing information and self-management skills is a key factor in reducing side effects and improving their quality of life [18], especially for the parents who usually do not have sufficient knowledge of the disease, treatment, and symptom management [19].

The results of studies specifically examining the role of educational interventions among children with acute lymphocytic leukemia (ALL) and their parents indicated that information provision and increasing parents’ knowledge about the disease greatly supported the family, enhanced parents’ ability to take care of their children, and considerably improved the family’s quality of life [20-23]. Consequently, patient education and the provision of information are major responsibilities of health care providers during the treatment period [24]. However, factors such as the multiplicity of tasks and limited time for education are the primary barriers to the full exercise of this responsibility [25,26]. As a result, they cannot spend enough time on patient education [27], and most of this instruction is provided by nurses via educational pamphlets or in-person sessions [20,28], which alone does not greatly affect health-related behaviors and outcomes [29]. For instance, approximately 40% to 80% of the information provided orally to patients is immediately forgotten, and half of the information is probably recalled incompletely or erroneously [30]. Using pamphlets does not seem to be a good method because it eliminates interactions and provides the same level of information for people with different levels of literacy and needs [31]. Therefore, there is a need for new methods to empower and enhance the knowledge of this group [32].

One such method is self-management, which is currently one of the best methods for cancer management [33]. Self-management emphasizes the role of education in preventive and therapeutic care activities [16]; highlights the role of the patient or caregiver in symptom identification, assessment, and reporting to health care providers; and helps the adoption of suitable measures for symptom prevention or control [34,35]. Usually, self-management interventions are implemented with the participation of health care specialists [16]; therefore, with the participation of clinical specialists, patients, and families, they can enhance patients’ knowledge and empower them to achieve therapeutic goals in different stages of treatment [33,35]. Studies show that even short periods of self-management education provided by health care specialists have positive effects on clinical outcomes [25] and increase the patient’s ability to control their chronic condition, thereby improving their quality of life [33,36]. An important feature of self-management interventions compared with conventional patient education programs is the customization feature that makes it suitable for use by every person [37].

This feature is made possible using information and communication technology tools such as smartphones [38]. The unique features of smartphones, such as accessibility, internet connectivity, and supported third-party apps, have extended their use in different fields, especially in the health care domain [39,40]. These features offer new opportunities to support patients with chronic conditions and provide them with information, education, and self-management skills. The World Health Organization introduces mobile health (mHealth) as a “domain of digital health aiming to provide or receive health-related information and services by using mobile communication and portable devices, e.g., cell phones, patient monitoring devices, personal digital assistants, and other wireless devices” [41,42]. The use of mHealth provides an opportunity for children and their families to receive information and education without visiting health care centers and specialists, control their condition through self-management, and enhance their quality of life [43,44].

Although the successful implementation of self-management programs requires the participation of all 3 groups, the results of a scoping review conducted in the requirement analysis stage revealed that numerous studies have been conducted in different countries, which developed mHealth interventions to support children with cancer and their families [45]. However, only a few studies engaged all stakeholders and developed self-management systems specifically to support children with ALL and their parents, which is the most prevalent type of pediatric cancer.

For instance, Wang et al [46] designed the Care Assistant app that provides access to clinical information and economic and social support for parents of children with ALL. In fact, the main users and audience of the app were caregivers. Heneghan et al [47] also developed a smartphone app to enhance medication adherence in adolescents with ALL. This app includes a list of relevant drugs and reminders for taking drugs.

### Objectives

There is no comprehensive solution that can garner the cooperation and participation of all three groups (children, parents, and oncologists) and address their different needs. Therefore, in line with the World Health Organization’s strategy in terms of performing interventions to increase the survival rate of children with cancer in LMICs by 2030 [48], we intend to develop and test a self-management system that provides information about ALL and self-management skills and facilitates interaction with oncologists for children and their parents.

## Methods

### Setting

This study was conducted between January and February 2020 at MAHAK’s Pediatric Cancer Treatment and Research Center. MAHAK is a highly specialized pediatric cancer hospital in Iran. This center has 100 hospital beds and covers >25,000 children with cancer from all over the country (Iran) and neighborhood countries. According to the latest official reports, >6000 children with cancer receive chemotherapy at this center annually [49].

### Participants and Recruiting Method

All participants were recruited from MAHAK’s Pediatric Cancer Treatment and Research Center in the north of Tehran (Iran). We used a banner that provided information about the study (in the MAHAK outpatient chemotherapy clinic) to recruit interested individuals in this study. A total of 35 individuals (n=24, 69% parents and n=11, 31% children) volunteered to participate in the study. However, only 63% (15/24) of parents and 73% (8/11) of children were eligible to participate. The inclusion criteria for children were having ALL, being diagnosed, being aged at least 7 years old (able to express the disease and the associated problems), and being under treatment for more than a year. The inclusion criteria for the parents were being literate in Persian and having at least one child with a diagnosis of ALL who was receiving treatment for 1 year. These purposive samples of children with ALL and their parents allowed for the aggregation of data related to the development and primary evaluation of this study. Health care providers (oncologists) were also recruited from this center. The inclusion criterion for this group was having at least 10 years of clinical experience in the oncology department. Children were excluded if they were illiterate, were patients with end-stage cancer, experienced mental health problems, and were unable to use a smartphone. Parents were excluded if they did not have the ability to work with a smartphone, did not have reading and writing literacy, or were unwilling to provide informed consent.

### Ethical Considerations

This research received ethical review approval from Shahid Beheshti University of Medical Sciences (IR.SBMU.RETECH.REC.1396.1316). All focus group (FG) meetings took place at the MAHAK hospital and were led by a team member (AM) with experience in conducting FGs. To avoid the dominance of professional feedback and blend the knowledge and expectations of other stakeholders, the identification of user needs was examined separately from 3 perspectives. For each FG, approximately 5 participants were invited. To attend the FG sessions, these children came to a meeting place with their parents. The children and their parents signed informed consent forms before each FG. For children, appropriate written and verbal information about the study and FG was provided, and informed consent was obtained from their parents to participate in the study. They also completed brief demographic questionnaires that provided information about age and sex, as well as their disease-related information.

### Study Design

#### Overview

**Figure 1:**
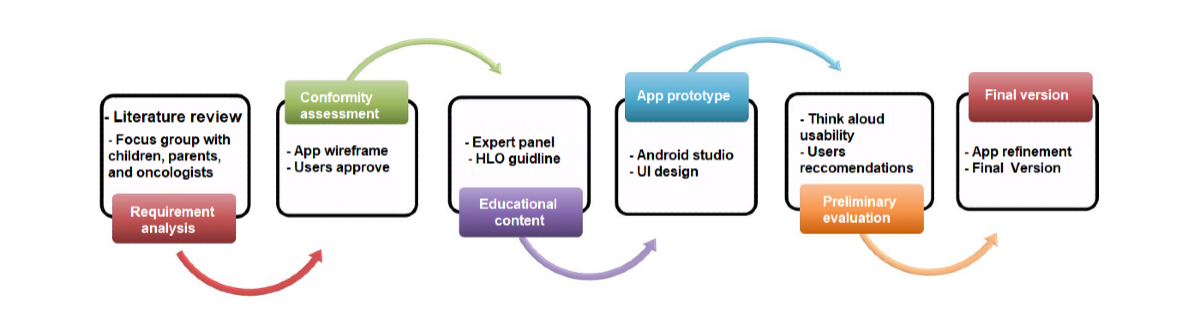
User-centered design process in this study [16]. HLO: Health Literacy Online; UI: user interface.

We adopted the user-centered design approach (Figure 1), in which all the stages of design, development, and evaluation were performed with the participation of the end users [50,51]. On the basis of this approach, the system was designed with the participation of children with ALL, their parents, and a multidisciplinary team (software engineers, medical informatics specialists, oncologists, pediatricians, and psychologists) in the following stages. Moreover, a qualitative methodology (FGs) was applied to identify the most relevant requirements from the users’ perspective.

#### Requirement Analysis

There was no standard regarding the components and content of a self-management system for cancer; thus, we conducted a review to identify the common features and components of such apps for children and adolescents with cancer and their families. The FG method was then adopted to identify user requirements. A total of 3 separate meetings (1 session per group) were held for children with ALL, their parents, and specialists. These meetings were held in the conference hall located on the fourth floor of the MAHAK. The sessions lasted 40 minutes for children (based on the FG guidelines for children) [52,53] and 90 minutes for adults and specialists. All sessions were recorded, and qualitative data were analyzed using thematic analysis. Descriptive statistics were used to characterize the study participants.

#### Conformity Assessment

Conformity assessment was a team effort with the collaboration of end user representatives and design team members. On the basis of a series of high-level requirements identified in the previous step, we developed wireframe prototypes (the app’s user interface [UI] version, which is not executable and made by sketcher software). Wireframes were examined by 38% (3/8) of children with ALL (aged 7-14 years) and 20% (3/15) of parents. The aim of this session was to ensure the adaptation of the initial design to the users’ identified needs.

#### Preparing Educational Content

To prepare the educational content, we used guidelines provided by the Cancer Council [54], American Cancer Society [55], Cancer Care Ontario [56], and Children’s Cancer and Leukemia Group [57], which are reputable organizations and institutions active in the pediatric cancer field. After being reviewed by 3 oncologists, a pediatrician, and a pediatric psychologist, the selected content was translated (to Persian) and organized based on the Health Literacy Online guidelines. This guideline assists in the development of educational content that is understandable by people with different health literacy levels [58,59].

#### App Prototyping

The prototype was developed after requirement analysis, conformity assessment, and preparation of educational content. This version almost resembled the final version, and users were able to experience the app and communicate with a UI.

#### Preliminary Evaluation

In the next stage, to ensure the accurate performance of the app, a preliminary evaluation was performed using think-aloud (TA) usability testing. An important advantage of using TA in the preliminary stage of design is the identification and correction of UI problems before developing the final version [60].

This evaluation was performed with 33% (5/15) of parents and 63% (5/8) of children with ALL, and 33% (5/15) of parents who were willing to participate in the study and had an Android smartphone. In this meeting, the researcher explained how the app works and is used. Each user then performed a number of predetermined tasks on the app while orally expressing their thoughts about functionality, problems, ideas, and exceptions. The researcher then recorded these ideas.

#### App Development

A final version was developed based on the results obtained from the preliminary evaluation. To ensure that the final version of the CanSelfMan app (a cancer self-management app) meets user expectations, an appropriate evaluation must be undertaken.

## Results

### Stage 1: Requirement Analysis

First, a review study was conducted as there were no guidelines or standards on the features and components of self-management apps for children with ALL. On the basis of the results, the modules of symptom evaluation, disease information, communication with specialists, and reminders had the highest frequency [45]. For requirement analysis, the results were then discussed in FG sessions with parents and children with ALL. Approximately 21% (5/24) of parents were excluded as only 2 months had passed since their child was diagnosed, 13% (3/24) as their child was in the end stages of cancer, and 4% (1/24) as they did not have a smartphone. Additionally, 27% (3/11) of children were excluded from the study as they were in the end stage of cancer. The first FG session involved volunteering parents (10/15, 67%; 6/10, 60% women). The parents’ mean age was 35 (range 28-43) years, with an education level above high school diploma. The second FG session involved 63% (5/8) of children with ALL (Table 1). The third FG session was held with 6 specialists (n=3, 50% pediatric oncologists; n=1, 17% radiotherapy oncology specialist; n=1, 17% pediatrician; and n=1, 17% pediatric psychologist), and their ideas about the self-management system feature were collected.

**Figure 2:**
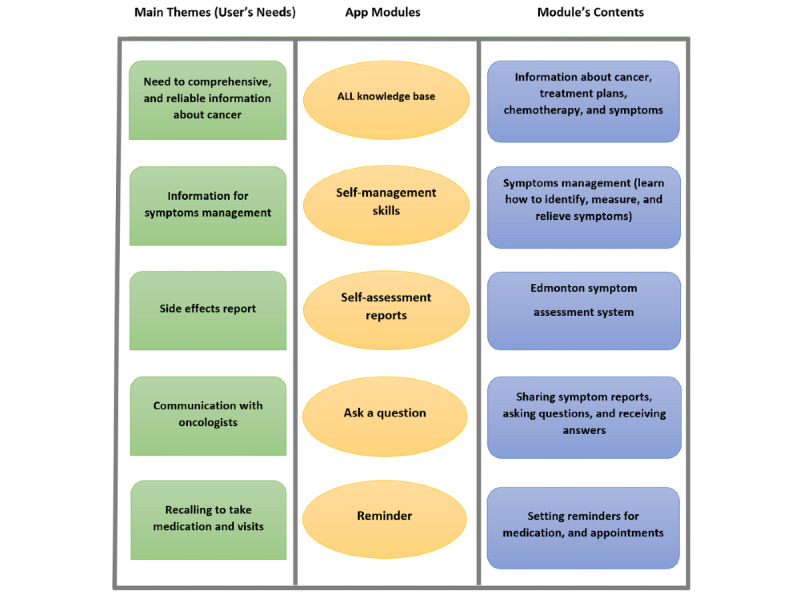
Focus groups extracted main themes, app module, and their content [61]. ALL: acute lymphocytic leukemia.

To identify the main themes, all sessions were recorded, transcribed, and thematically analyzed independently using HM and AM. Interestingly, the main themes obtained through thematic analysis were almost the same as those obtained from a review study conducted in the initial stage. Based on the themes identified from the FG sessions, the app modules were determined (Figure 2).

**Table 1:** Demographic characteristics for children and parents.

Study phases			Focus group sessions					Think-aloud session		
			Children (n=5)		Parents (n=10)		Children (n=5)			Parents (n=5)
Age (years), mean (SD; range)			9 (1.9; 7-14)		35 (2.9; 28-43)		11 (1.1; 7-14)			32 (2.8; 25-46)
**Gender, n (%)**										
	Female	3 (60)		6 (60)		2 (40)			3 (60)	
	Male	2 (40)		4 (40)		3 (60)			2 (40)	
**Marital status, n (%)**										
	Married parent	N/A^a^		4 (80)		N/A			5 (100)	
	Single parent	N/A		0 (20)		N/A			0 (0)	
**Education level, n (%)**										
	Secondary school	N/A		1 (10)		N/A			1 (20)	
	Diploma	N/A		5 (50)		N/A			2 (40)	
	Bachelor’s degree and above	N/A		4 (40)		N/A			2 (40)	

^a^N/A: not applicable.

### Stage 2: Conformity Assessment

**Figure 3:**
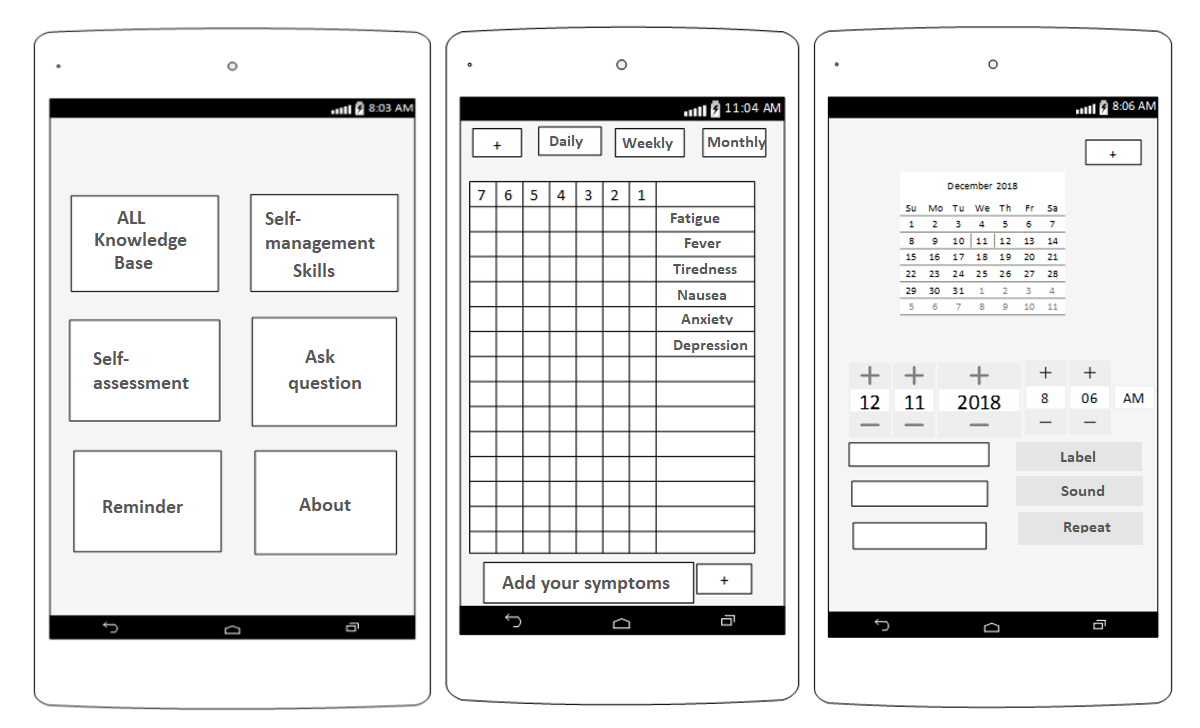
Example of app wireframe for conformity assessment sessions. ALL: acute lymphocytic leukemia.

These requirements were subsequently used to develop wireframe prototypes and technical requirements for the development phase. A total of 3 children with ALL (n=2, 67% girls; mean age 10, SD 1.4 years) and 3 parents (n=2, 67% men; mean age 39, SD 1.8 years) as representatives of the end users (selected from FG participants) were selected and took part in this session. In this session, two members of the design team (a software engineer and a medical informatics specialist) attended as well. The aim of this session was to confirm the initial design based on the users’ views and modify it if necessary. In this 45-minute session, a list of the app’s functionalities was first provided. Finally, for a better understanding of the user requirements, the wireframe was presented to the participants (Figure 3). At the end of the session, all the participants mentioned that the prototype was based on the requirements identified in the FG sessions.

### Stage 3: Preparing Educational Content

In the next step, a 90-minute expert panel was held with oncologists (4 oncologists with 15 years of experience, a radiotherapy oncology subspecialty with 14 years of experience, and a pediatric subspecialty with 11 years of experience) and a medical informatics specialist to determine the educational content. After review by this group, 4 main guidelines were selected (the Cancer Council and the American Cancer Society’s guidelines for providing information and the Cancer Care Ontario and Children’s Cancer and Leukemia Group guidelines for preparing self-management content). Selected content was then translated (to Persian) and organized based on the Health Literacy Online guidelines. This guideline assists in the development of educational content that is understandable by people with different health literacy levels [58,59].

### Sage 4: App Prototyping

In stage 4, a prototype was implemented using the Java programming language and SQLite database in Android Studio. For the web-based version, we used the PHP programming language and MySQL database. The first prototype had five modules: information about ALL, self-management tips, self-assessment form, ask questions, and reminder function. This version was fully executable.

### Stage 5: Preliminary Usability Evaluation

The first prototype was evaluated using the TA method with 10 representatives of the user groups (n=5, 50% children, with n=2, 40% girls; n=5, 50% parents, with n=3, 60% women). This method was performed individually. Before starting the test, in 5 minutes, some explanations about the app and TA evaluation were provided by HM. The app was then installed on the user’s smartphone, and some predetermined tasks were performed. At the same time, the user orally provided comments on the problems, ideas, functionality, and expectations. The researcher recorded each user’s comments. Some suggestions and problems expressed by children and parents at this stage are provided in Textbox 1.

**Think-aloud primary evaluation results.**

**Think-aloud comments**

- “I wish we could select a profile picture.”
- “I worked with a game app before; it had a cartoon character. I wish this app had a cartoon character that I could see on every page. It would look more attractive.”
- “In the symptom evaluation part, score smileys can’t be seen for symptoms of the last row. You have to scroll up every time to see them. I wish they were fixed so that it’d be easier to fill out the questionnaire, and you wouldn’t have to scroll up.”
- “In the evaluation part, it’d be more practical if you could view the results in the form of charts and send them to a specific Oncologist.”
- “The font is too small and illegible in the information about the disease part; I wish we could change the font size.”
- “In my favorites part you can’t remove an item from the list. When I added a topic to the list, I couldn’t remove it later.”
- “In the reminder part, you can’t choose all days of the week. You can select the minutes only in quarters. You can’t exit the app.”

### Stage 6: Develop Final Version

**Figure 4:**
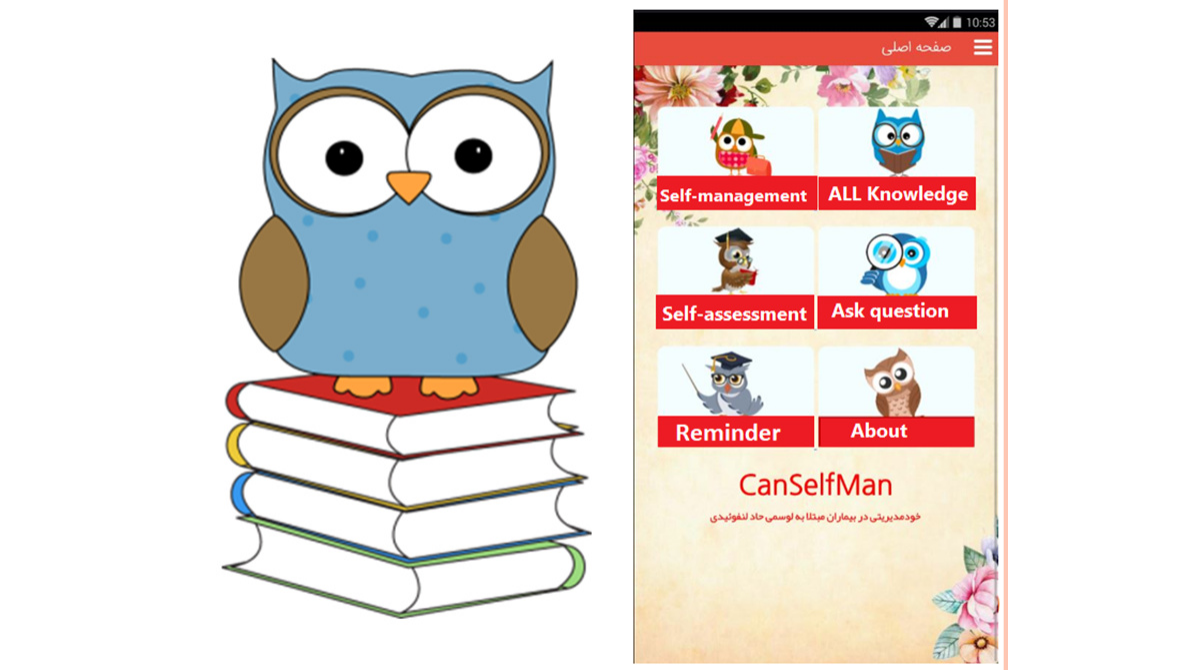
Owl character and the CanSelfMan (cancer self-management) app main page.

Immediately after the preliminary evaluation, modifications were made, and the final version of the CanSelfMan app was developed. The app had 2 different versions for children and parents and a web-based dashboard for oncologists (Multimedia Appendixes 1 and 2). The CanSelfMan UI was specifically designed using gamification elements to provide a better user experience. On the basis of the user’s suggestion, an owl character was selected as the app symbol (Figure 4). This character was selected based on the Dove, Owl, Peacock, and Eagle Psychological Test that divides people based on their characteristics into four groups: dove (symbolizing peace and kindness), peacock (extroverted and showy), owl (logical and smart), and eagle (courageous and decisive) [62]. The final version had five modules: ALL knowledge base, self-management tips, self-assessment report, ask questions, and reminders. The module provides explanations about the app and how to use it.

**Figure 5:**
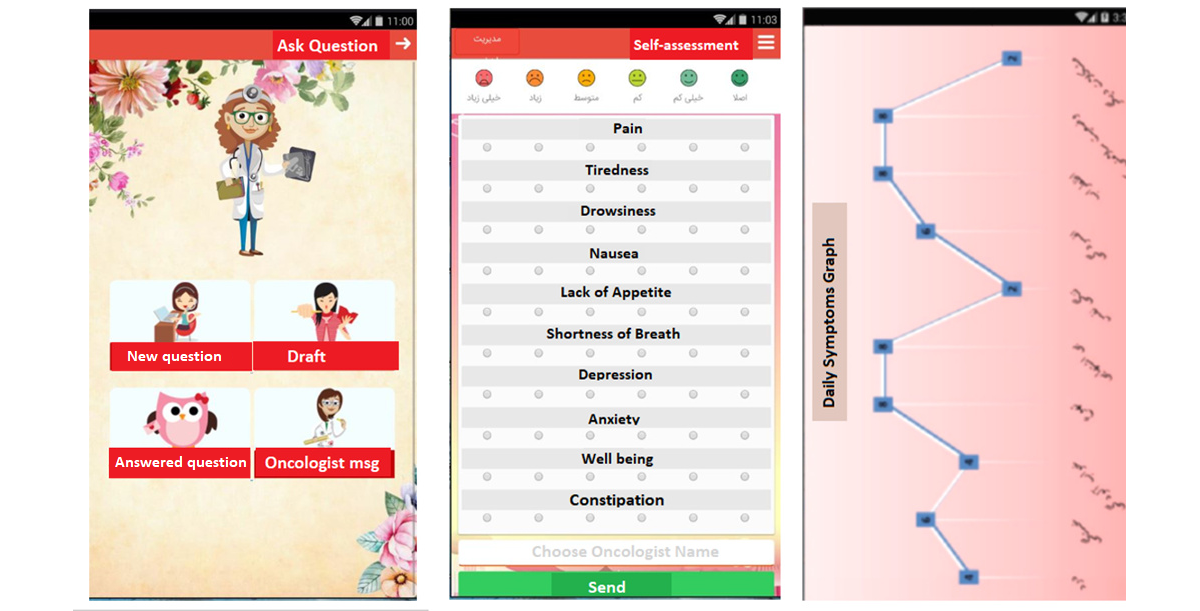
The CanSelfMan app (cancer self-management) screenshots of (ask question and self-assessment module).

The ALL knowledge base provides information on the definition, etiology, diagnostic methods, medications, and common treatment protocols for ALL. The self-management module included information on identification of symptoms, evaluation, and control via pharmaceutical and nonpharmaceutical methods, physical exercise, and nutritional information. A standard Edmonton Symptom Assessment System–Revised questionnaire was used in the self-assessment report module. This questionnaire (on a 10-point scale) is commonly used in cancer care centers as a tool for the self-evaluation and reporting of symptoms by patients. After consulting with oncologists for legibility and to facilitate the completion of the form by children, a visual equivalent was used for the options for easier use and response [63,64]. In this part, the user could complete the evaluation form and view the results via graphic charts, send them to the oncologists, or present them to oncologists during in-person visits. The next module was *ask a question*, which enabled direct communication between children or parents and the oncologist (Figure 5). The users could ask their questions by completing and sending the form and choosing the oncologist’s name. The important point in this module is the response time to the questions; this time was set at a maximum of 24 hours, as agreed by the oncologists. Moreover, medical recommendations based on the reported symptoms could be sent. The next module was the *reminder* module. Users could create reminders for different topics (taking medications and oncologist’s appointments). The app notified the user at specified times.

The web-based version of the app provided a dashboard to view the reports and presented questions and answers to the patients and parents. This version was designed for oncologists. Its main page displayed the questions and reports received from the patients. The oncologist could select a question and answer it. In self-report management, the oncologist could view the reports sent by the users and, if necessary, make suggestions or send messages.

## Discussion

### Principal Findings

Most new technologies are not popular among users as they do not participate in them or consider their needs [65]. On the basis of studies, the main reason for the lack of use of mHealth apps is that they are developed without the participation of end users; therefore, in most cases, the outcomes are not compatible with user requirements and will probably not be used by them [47,66]. To resolve this problem, a user-centered design approach was adopted, which is an evidence-based approach based on the understanding of users’ needs by involving their participation in all stages of the design and development process [66,67]. This method plays a key role in user interest and acceptance and increases the chances of intervention effectiveness [68]. Therefore, we used this method to develop the CanSelfMan app. All three user groups (children, parents, and oncologists) participated in the stages. Similarly, Ben-Zeev et al [69] developed a self-management system by collaborating with a multidisciplinary team of oncologists, nurses, software engineers, and end users.

In this study, we used a qualitative approach (FG) to identify system requirements and primary evaluations by collecting narrative information, including people’s experiences and feelings. This approach typically uses small sample sizes to investigate users’ expectations and beliefs regarding the natural environment rather than looking for generalizable outcomes for larger samples [70]. There is no clear standard for the number of children in an FG; however, generally, between 4 and 5 participants seems enough to acquire sufficient data [53,61].

For example, to design Sisom (an app for symptom reporting by children with cancer and direct communication with health care specialists), Arvidsson et al [51] involved children aged 6 to 12 years. Similarly, to design the Care Assistant app, Wang et al [46] used FG sessions to identify parents’ needs and provide access to clinical information and economic support. In the preliminary stages of designing the CanSelfMan app, to ensure that the final version meets user needs, we used wireframes before the final UI design to confirm the user requirements. Presenting a preliminary UI design provides users with an overall view of the final product, which may minimally differ from what the users had seen through the wireframe. Therefore, users’ participation in confirming/providing suggestions about the preliminary design garnered their trust and reduced the risk in accepting the final product [71]. After confirming the preliminary design, a prototype was developed that contained all the specified modules and had a full UI. The TA usability test was performed at this stage. It is one of the most important evaluation methods in the preliminary stages that identifies possible defects in the UI design and program logic (product) before developing the final version [60,72].

In this study, we used the TA method to evaluate the CanSelfMan app prototype with the participation of two user groups (5 children and 5 parents). On the basis of the TA results, we developed the final version of the CanSelfMan app. Similarly, Baau and Markopoulos [73] compared the use of TA and poststudy interviews with children and found that the TA method identified more usability issues than the interviews. Owing to the labor-intensive nature of the TA approach, the sample size was typically small. However, small numbers do not indicate a small data set, and small sample sizes can still yield valid information [74]. Nielson [75] suggested that samples as large as 5 participants would provide adequate information using this method.

To develop an app for pain control and evaluation in adolescents with cancer, Jib et al [76] used this method for the preliminary evaluation of the app and collection of users’ feedback. To attract users and motivate them to use the app, we used warm colors and cartoon characters. Using visual and gamification elements can lead to increased user motivation and reduce the sense of boredom, especially in children and adolescents [32]. The use of an owl cartoon character in different parts of the UI and the creation of avatars followed the same goal. In a similar study to develop an app for pain management in children and adolescents with cancer, Stinson et al [77] used graphic elements and warm colors to increase children’s cooperation. This technique increased the use of the app and completion of the pain reporting forms [77].

The goal of designing this app was to support children with ALL and their parents in dealing with cancer and enable their communication with health care specialists. The end users of this app are classified into three general groups: (1) children aged >7 years who can use the app independently, (2) children aged between 5 and 7 years who cannot use the app independently and need the help of parents, and (3) parents who can use the app and its features independently or act as a proxy for using this app for their children aged <5 years. A web-based version was also developed for oncologists, which provided interactions with children and parents. This interaction included questions and answers about the disease and treatment or requests for changing the in-person appointment dates. Self-assessment, symptom reporting to the oncologist, and receiving feedback create an information flow that increases the interaction between patients or parents and oncologists [78]. This is more important when interacting with children because of the difficulty in communicating with them and their lack of verbal cooperation about the symptoms they experience [79]. Symptom reporting and evaluation are a common part of cancer self-management apps. Parents’ reports are a proper source for the symptom evaluation and reporting of children aged <5 years, children with cognitive problems who have developmentally lower levels of understanding of the disease and symptoms, or children who cannot perform self-reporting because of their conditions [62]. Even when children cannot report their condition, parents’ reports can offer a complementary view about the child’s condition as parents have accurate knowledge about the child’s health and are closely involved in the process of medical decision-making; however, their reports should not lead to the ignorance of children’s views [80,81]. Therefore, based on the important role of the parents in taking care of children, and because of the limited abilities of children, especially when they are aged <8 years, any intervention whose target group is children should also involve parents in the intervention design as an inseparable part.

### Strengths, Limitations, and Suggestions

In this study, we developed the CanSelfMan app in 2 different versions for children aged >7 years, and another version for their parents can be used for children aged <7 years. One of the highlights of our study was the participatory design approach to developing the app. All stages of app development and evaluation were performed with the participation of end users. This approach, owing to the focus on users and their needs, can increase user acceptance and user satisfaction with the final product. Another key strength of our study was the presence of people from different academic disciplines or professional specializations in the design team (software engineers, medical informatics specialists, oncologists, pediatricians, and psychologists). This variety of expertise and differing views benefited the study and helped us cover different aspects of the system and respond to users’ needs from a broader and more diverse perspective. In addition, most of the mHealth interventions focused on children aged >8 years, and few studies targeted those aged <8 years. A reason for this might be the uncertainty about children aged ≤7 years regarding their ability to understand information about cancer and symptom assessment. However, evidence has shown that most children, aged as young as 5 years, can fill in symptom assessment questionnaires alone or with their parents’ help [81].

The main limitation of this study was related to the small number of participants in the requirement analysis and evaluation phases. On the other hand, the CanSelfMan app was developed based on the requirement analysis of children with ALL and their parents in only 1 cancer treatment center. Therefore, the generalization of the study findings is limited because of the fact that the data were gathered from a single facility with a limited age group of patients. We accepted that this may have increased the risk of bias. It is likely that the participants’ views in this study are not necessarily those of all children with ALL or their parents. Therefore, additional research is needed to examine more children’s and parents’ perspectives before wider implementation. However, as the purpose of this study was to develop a self-management system and not to investigate the effect of this app, the small number of participants in this process was not an obstacle to achieving this goal. We conducted only a primary usability evaluation; thus, future studies should include a larger sample size with a wider age range of participants and allocate more time for participants to test the UI and evaluate usability, which will be the focus of our future study. In addition, the clinical findings of this intervention have not yet been studied; thus, further clinical trials are required to demonstrate the efficacy of this product in relation to routine care.

### Conclusions

The use of mHealth can facilitate access to accurate information about cancer in patients and their families. To access these services, users should only have a smartphone and little knowledge about the use of these tools. Despite these confirmed advantages, most mHealth studies have been conducted in developed and high-income countries, and the share of underdeveloped or developing countries in these studies is minimal. Meanwhile, a rise in the incidence rate of childhood cancer is predicted in future years (which will mainly occur in LMICs). Therefore, we developed a CanSelfMan system (smartphone app + web-based dashboard) to enhance self-management skills in children with ALL and their parents or caregivers. The CanSelfMan app can support these groups by providing access to reliable information and symptom management skills and facilitating communication between child/parents and oncologists. It can also be used in specialized cancer centers, especially LMICs, to increase access to these services. Future evaluation studies need to investigate the program’s effectiveness and cost-effectiveness.

## Appendix

Multimedia Appendix 1 The CanSelfMan (cancer self-management) app web version tutorial.

Multimedia Appendix 2 The CanSelfMan (cancer self-management) app tutorial.

## Abbreviations

ALL: acute lymphocytic leukemia
FG: focus group
LMIC: low- and middle-income country
mHealth: mobile health
TA: think-aloud
UI: user interface
